# A Review of Default Mode Network Connectivity and Its Association With Social Cognition in Adolescents With Autism Spectrum Disorder and Early-Onset Psychosis

**DOI:** 10.3389/fpsyt.2020.00614

**Published:** 2020-06-25

**Authors:** Aarti Nair, Morgan Jolliffe, Yong Seuk S. Lograsso, Carrie E. Bearden

**Affiliations:** ^1^ Department of Psychiatry and Biobehavioral Sciences, Semel Institute for Neuroscience and Human Behavior, University of California, Los Angeles, Los Angeles, CA, California; ^2^ Graduate School of Professional Psychology, University of Denver, Denver, CO, United States; ^3^ Division of the Humanities and Social Sciences, California Institute of Technology, Pasadena, CA, United States; ^4^ Department of Psychology, University of California, Los Angeles, Los Angeles, CA, United States

**Keywords:** functional connectivity, default mode network, social cognition, autism spectrum disorder, early-onset psychosis

## Abstract

Recent studies have demonstrated substantial phenotypic overlap, notably social impairment, between autism spectrum disorder (ASD) and schizophrenia. However, the neural mechanisms underlying the pathogenesis of social impairments across these distinct neuropsychiatric disorders has not yet been fully examined. Most neuroimaging studies to date have focused on adults with these disorders, with little known about the neural underpinnings of social impairments in younger populations. Here, we present a narrative review of the literature available through April 2020 on imaging studies of adolescents with either ASD or early-onset psychosis (EOP), to better understand the shared and unique neural mechanisms of social difficulties across diagnosis from a developmental framework. We specifically focus on functional connectivity studies of the default mode network (DMN), as the most extensively studied brain network relevant to social cognition across both groups. Our review included 29 studies of DMN connectivity in adolescents with ASD (Mean age range = 11.2–21.6 years), and 14 studies in adolescents with EOP (Mean age range = 14.2–24.3 years). Of these, 15 of 29 studies in ASD adolescents found predominant underconnectivity when examining DMN connectivity. In contrast, findings were mixed in adolescents with EOP, with five of 14 studies reporting DMN underconnectivity, and an additional six of 14 studies reporting both under- and over-connectivity of the DMN. Specifically, intra-DMN networks were more frequently underconnected in ASD, but overconnected in EOP. On the other hand, inter-DMN connectivity patterns were mixed (both under- and over-connected) for each group, especially DMN connectivity with frontal, sensorimotor, and temporoparietal regions in ASD, and with frontal, temporal, subcortical, and cerebellar regions in EOP. Finally, disrupted DMN connectivity appeared to be associated with social impairments in both groups, less so with other features distinct to each condition, such as repetitive behaviors/restricted interests in ASD and hallucinations/delusions in EOP. Further studies on demographically well-matched groups of adolescents with each of these conditions are needed to systematically explore additional contributing factors in DMN connectivity patterns such as clinical heterogeneity, pubertal development, and medication effects that would better inform treatment targets and facilitate prediction of outcomes in the context of these developmental neuropsychiatric conditions.

## Introduction

Autism spectrum disorder (ASD) and schizophrenia are heterogeneous conditions that share several phenotypic and genomic features (1–4). For instance, deficits in social interaction, emotional reciprocity, pragmatic speech, and theory of mind (ToM) are postulated to be central to both disorders (5). While early detection and clinical diagnosis of both disorders has improved over the past decade, frequent challenges still arise in differential diagnosis (e.g., in the event of later diagnosis of ASD) especially if predominant symptoms for both involve social difficulties and unusual social thinking (2, 6). Recent behavioral studies of adults with ASD and schizophrenia highlighted not only the similarities but also some divergent patterns of social impairments in the two disorders—with ASD characterized by lower social motivation, poorer social reciprocity, and undermentalizing, and schizophrenia characterized by greater reciprocity but poor expressiveness (7, 8). Moreover, these social impairments are associated with difficulties in the work setting (9, 10), social relationships (11, 12), and overall reduced quality of life (13, 14) across both groups. While several studies have demonstrated genetic overlap between ASD and schizophrenia (2–4, 15), the neural mechanisms underlying the pathogenesis of the social impairments observed in these disorders are still not well understood. Given the public health significance of social disability and social isolation (16), it is crucial to explore the neurobiological mechanisms underlying social deficits across both groups, as well as to understand how they relate to real-world behaviors. Exploring the shared and distinct neural underpinnings in ASD and schizophrenia could advance our understanding of social cognitive deficits across these conditions, which will ultimately help better inform treatment. Although antipsychotics have been shown to be effective in reducing positive symptoms in schizophrenia, they are not effective in addressing the devastating social disability associated with the disorder which contributes to chronic functional impairment (17, 18). It is thus imperative to identify behavioral interventions for children and adolescents that have already shown promise in other clinical groups such as ASD. By enhancing our understanding of the neurobiological underpinnings of social impairments in ASD and how they compare to those observed in schizophrenia, we will be able to refine treatment targets and predict outcomes for each group. Hence, here we conduct a narrative review of the existing neuroimaging literature on functional connectivity of a key social brain network (default mode network; DMN) in ASD and schizophrenia, in order to elucidate the shared and unique neural mechanisms underlying social impairments across diagnosis from a developmental framework.

Adolescence or youth (ages 10–24; 19, 20) is a particularly critical window for social development and thus is an important time to investigate neural mechanisms implicated in social functioning. Adolescence is a developmental period classified by gaining independence and autonomy from caretakers (21), with marked changes in identity, self-consciousness, and cognitive flexibility (22–24). As a part of this process of developing as an independent individual, there is typically an increase in peer-directed social interactions (21, 23, 25). As a result of this increase in sociality, adolescence is a time when the social demands change most dramatically, requiring individuals with social deficits to work harder. Prior research has shown that social deficits become even more apparent during this period as social contexts increase in complexity and pose higher social expectations (24, 26). Consequences of poor social skills include peer rejection or victimization, poor friendship quality, lack of social support, experiences of loneliness, poor academic and vocational outcomes, and the development of anxiety, depression, or other psychopathologies (27–29). For individuals with ASD, adolescence may be a particularly difficult developmental period as they are also experiencing increased motivation to engage with peers, yet likely have a greater awareness of their social deficits (30). For individuals with psychotic disorder, negative symptoms including social withdrawal, reduced communication, and general apathy often precede positive symptoms and are linked more strongly to poor prognosis (31–34). The fact that social deficits often precede full-blown positive symptoms in schizophrenia implies that there are likely neural changes occurring during adolescence that precede manifestation of psychotic symptoms in early adulthood. While social impairment is a hallmark of both ASD and psychosis, these difficulties may have distinct origins: for example, the hypo-hyper-intentionality hypothesis (1, 35) postulates that individuals with ASD may under-attribute intentions to others or “undermentalize”, whereas those with schizophrenia may over-attribute intentions to others or “overmentalize”, parlaying into symptoms of suspiciousness and paranoia.

In addition to contextual changes in the social environment, adolescence is also a period marked by significant neural changes, particularly in the prefrontal cortex, a major hub in several brain networks associated with social functioning (23, 36–41). Evidence suggests that while sensory and motor brain regions are fully myelinated within the first several years of an infants’ life, neurons in the frontal cortex continue to be myelinated through adolescence (21, 23, 40). This increased myelination as well as white matter density is coupled with decreases in cortical thickness and gray matter in social brain hubs in frontal and parietal lobes (38, 40). Additionally, synaptic pruning—the process of eliminating unused neural connections, and the reorganization of strengthened pathways—is occurring actively in the prefrontal cortex during puberty (23, 25, 42–44). As a result, adolescents experience a net decrease in synaptic density during this time (23) along with increased long-distance and decreased short-distance functional connections in the brain, indexing better network integration and segregation during this period (45, 46). Increases in functional activation of prefrontal cortex are also observed in typical adolescents compared to adults in response to social tasks (25, 38). Increased functional connectivity between prefrontal cortex and temporal brain regions during adolescence is also related to increased social information processing during this age (39).

Although the social brain is not a specifically defined network, there is general consensus in the literature that the medial prefrontal regions, the temporoparietal junction, anterior and lateral temporal regions, anterior insula, and the posterior cingulate cortex/precuneus subserve several crucial social functions (25, 40, 47). Of note, the aforementioned brain regions are all highly represented within the DMN—a large-scale brain network with hubs in the medial prefrontal cortex (mPFC), posterior cingulate cortex/precuneus (PCC), inferior parietal lobe (IPL), and temporal lobe structures (48–50). The DMN is one of the most extensively studied functional networks, and it shows substantial overlap with several other “social brain” networks such as the mentalizing network and emotion recognition network (47, 51, 52). It has been proposed that the DMN is specifically involved in self-referential thinking (53–56), thoughts about self versus others and theory of mind (50, 52, 57, 58), and autobiographical memory (55, 56). Prior studies investigating DMN connectivity in health adolescents have suggested that there is a strengthening of connectivity in this network with age, particularly between anterior and posterior hubs from childhood to late adolescence, indicating increased integration in typical development (36, 37, 41, 45). Additionally, these same studies have suggested that DMN connectivity with other functional networks such as the central executive network becomes sparse from childhood to late adolescence, suggestive of increased autonomy and segregation of the DMN from task-related networks in typical development.

Disrupted DMN functional connectivity has been implicated in several psychiatric conditions with associated social difficulties (47, 59–61), including ASD (62) and schizophrenia (63). With such a rich literature, investigation of DMN function in adolescence offers a window into understanding how these social brain regions are functionally connected, how they are altered in disorders affecting social function, and their relationship to real-world social deficits. Much of this existing literature on the social brain and DMN connectivity has, however, focused on children (for ASD) and adults (for schizophrenia/psychosis), with fewer studies focusing on adolescents. While ASD may be diagnosed earlier in life, there is evidence to suggest that functional connectivity patterns in individuals with this condition undergo substantial changes from childhood to adulthood, likely influenced by factors such as puberty and/or access to treatment interventions over the years (64). In contrast, the age of onset for psychotic disorder peaks in adolescence, but more subtle cognitive and socio-emotional disturbances are present in early childhood (65). It is posited that overt symptom onset of psychosis during adolescence may be related to underlying changes in brain connectivity patterns affected by hormonal changes and increased stress response during this period (40). Due to the importance of this developmental period for brain development in general, as well as the relevant changes to social contexts, examining brain networks implicated with social cognition such as the DMN in adolescence requires substantial attention to further our understanding of the shared and distinct neural mechanisms underlying the social cognition deficits present in each group.

Hence, the current article aims to further explore cross-sectional studies on DMN connectivity in ASD and early-onset psychosis (EOP) during the adolescent years. For this purpose, we reviewed the literature available through April 2020 in PubMed, Google Scholar, and PsycINFO on DMN connectivity in adolescents with ASD and/or EOP, using search terms including “default mode network, functional connectivity,” combined with “adolescence, autism, ASD, Asperger’s” or “psychosis, adolescent-onset psychosis, adolescent-onset schizophrenia, first-episode psychosis, early-onset psychosis, early-onset schizophrenia”. The initial literature search revealed 160 relevant studies in ASD and 46 in EOP. Studies were subsequently included in the review based on age-range spanning adolescence and patient groups meeting diagnostic criteria for either ASD or EOP. All included studies were also required to have a control group of typically developing adolescents. Additionally, we focused only on empirical studies that included either: 1) static resting-state analysis or dynamic functional connectivity (DFC) analysis that examines temporal variations in connectivity patterns across the duration of the scan (66–68), and 2) provided information about the directionality of their findings. The methods used for these studies included:

1. Traditional seed-based analysis (SBA), wherein the time-series from a seed-region are correlated with all other voxels in the entire brain or a mask of the DMN (69, 70).
2. SBA that quantify the amplitude of low-frequency fluctuations (ALFF), i.e., the magnitude of signal intensity of spontaneous fluctuations for a given brain region. In ALFF analyses, the time-series from a given seed-region are transformed into a frequency domain from which the power spectrum are obtained (70, 71).
3. Independent component analyses (ICA), a data-driven method wherein whole-brain signal are decomposed to identify spatially and temporally independent components. Software templates of the DMN are then used to identify components that correspond to this network (70).
4. Support vector machines (SVM) are data-driven supervised machine-learning methods using pattern recognition algorithms to automatically classify neuroimaging data into typical or atypical categories (72).
5. Self-organizing map (SOM) algorithm, a clustering analysis technique wherein voxels are organized on a two-dimensional matrix with each node representing clusters of voxels that are highly correlated and nodes that are closer together on matrix representing neural networks (72, 73).
6. Regional Homogeneity (ReHo), a voxel-based approach to measuring brain connectivity wherein the similarity between the time-series of a given voxel and its nearest neighbors within a network is evaluated (74).
7. Granger Causality Analysis (GCA), a statistical method that allows for prediction of causality between functional connectivity of two seed-regions/nodes from time-series data (75).
8. Network Homogeneity (NH), a voxel-wise measurement of homogeneity and cohesiveness of each voxel within a functional network that provides an index of network integrity (76).

Our final review included 29 studies of DMN connectivity in adolescents with ASD (mean age range = 11.2–21.6 years; see **Table 1** for demographic details), and 14 studies in adolescents with EOP (Mean age range = 14.2–24.3 years; see **Table 2** for demographic details). Our goal is to synthesize the findings of altered DMN connectivity from the existing literature for each clinical population within a developmental framework and discuss how the potential commonalities or differences in underlying neural mechanisms may relate to characteristic symptomatology. We conclude by providing some insights into gaps within the extant literature and highlighting future directions for research.

**Table 1:** Summary of demographic details and results for studies on DMN functional connectivity in adolescents with ASD.

Study	Sample Size	Age			Sex (F%)		IQ	Dataset	Analysis	Results for ASD group
	N (ASD/TD)	ASD Mean (SD)	TD Mean (SD)	ASD/TD						
77	30 (15/15)	15.7 (3)	17.1 (3.6)	6.7%/13.3%		ASD IQ=113.3 ± 15.0 TD IQ=117.1 ± 16.9			ICA	Underconnectivity within DMN hubs of precuneus (PCUN), and medial prefrontal cortex (mPFC).
78	31 (16/15)	15(1.45)	16(1.44)	12.5%/6.7%		No Full-Scale IQ ASD VIQ=114 ± 18.58 ASD NVIQ=117 ± 13.82 TD VIQ=113 ± 14.10 TD NVIQ=106 ± 12.53			SBA	Underconnectivity between posterior cingulate cortex (PCC) hub of DMN and 9 of 11 other DMN regions – retrosplenial cortex, and bilateral mPFC, superior frontal gyri (SFG), temporal lobe, parahippocampal gyri (PHG).
73	80 (39/41)	14(2.08)	15.3(2.4)	17.9%/19.5%		No Full-Scale IQ ASD VIQ=108.2 ± 19.04 ASD NVIQ=111.54 ± 15.97 TD VIQ=116.5 ± 13.34 TD NVIQ=105.4 ± 11.51			SOM	Underconnectivity between posterior hubs of DMN and right (r) SFG.
79	28 (14/14)	17.8(1.9)	17.7(1.8)	0%/0%		ASD IQ=116.9 ± 13.7 TD IQ=119 ± 9.6			SBA	Overconnectivity between anterior (a) MPFC hub of the DMN and right lateral parietal (rLP) seed.
80	50 (24/26)	14.9(1.4)	14.8(1.7)	25%/26.9%		ASD IQ=107.3 ± 16.9 TD IQ Not Reported			ICA	Underconnectivity between anterior and posterior DMN subnetworks.
81	964 (447/517)	16.6(8.1)	16.9(7.56)	11.4%/17.6%		ASD IQ=105 ± 17.4 TD IQ=112 ± 13.3		ABIDE	SBA	Reduced left lateralization in connectivity between PCC hub of DMN and language regions (Wernicke’s area).
82	115 (71/44)	12.3(3.1)	12.2(3.8)	0%/0%		ASD IQ=97.8 ± 19.7 TD IQ=117.2 ± 9.7			SBA	Mixed results with underconnectivity between left PCC hub of DMN and left (l) MPC, right inferior temporal gyrus (rITG), and bilateral angular gyri (AG). In contrast, overconnectivity between PCC hub and inferior parietal lobule (IPL), superior parietal gyri (SPG), SFG, middle frontal gyri (MFG) and precentral gyri (PreCG).
83	39 (22/17)	13.8(2.0)	12.8(3.6)	23.5%/13.6%		ASD IQ=107.8 ± 18.7 TD IQ=107.8 ± 14.3			ICA/SBA	Mixed results with local overconnectivity in dorsal (d) anterior cingulate cortex (ACC) but underconnectivity between dACC and PCC/PCUN hub of DMN.
84	56 (28/28)	13.71(1.79)	14.01(1.74)	17.9%/17.9%		ASD IQ=103.57 ± 15.45 TD IQ=105.18 ± 9.90		ABIDE	ICA	Children showed within-DMN overconnectivity but not adolescents; Adolescents showed underconnectivity between DMN and subcortical/insular network.
85	75 (37/38)	13.9(2.6)	13(2.6)	13.5%/21%		No Full-Scale IQ ASD VIQ=105 ± 19.3 ASD NVIQ=104 ± 16.0 TD VIQ=107.8 ± 11.8 TD NVIQ=107.5 ± 12.5			SBA	Mixed results with underconnectivity within mPFC and PCC hubs of the DMN and overconnectivity between PCC and right ventrolateral prefrontal cortex (rVLPFC) and rIPL, mPFC and rVLPFC, and lAG and right dorsolateral prefrontal cortex (rDLPFC) and rIPL.
86	185 (90/95)	13.1(3.3)	13.2(3.1)	0%/0%		Not Reported		ABIDE	SBA	Overconnectivity between bilateral mPFC hub of DMN with bilateral IPL and right anterior insula (AI).
87 (Study 1)	152 (76/76)	16.1(4.9)	15.8(4.5)	11.8/15.8%		**Study 1:** ASD IQ=106.6 ± 18.1 TD IQ=108.1 ± 12.4 **Study 2:** ASD IQ=106.3 ± 18.0 TD IQ=109.5 ± 11.1		ABIDE	DFC/SBA	Greater temporal variability across windows, as well as predominant underconnectivity within DMN regions such as PCC with mPFC, ACC, and right hippocampus, and mPFC with lLP.
87 (Study 2)	64 (32/32)	14.3(2.4)	13.5(2.7)	12.5%/15.6%						
88	134 (51/40) 43 Unaffected Siblings	ASD M 14.5(1.7) ASD F 14.5(2.0) M Sib 15.0(2.1) F Sib 14.6(2.2)	M 14.8(1.7) F 15.3(5.3)	31.4%/50% Siblings 69.8%		ASD M IQ=108 ± 16.1 M Sib IQ=113.5 ± 11 ASD F IQ=97.6 ± 10.7 F Sib IQ=112 ± 9.6 TD M IQ=114 ± 11.4 TD F IQ=110.7 ± 10.9		ABIDE	SBA	Underconnectivity within-DMN network in both males and females with ASD even compared to unaffected siblings.
89	46 (22/24)	13.1(3.1)	15.4(1.6)	31.8%/29.2%		ASD IQ=95.2 ± 22.1 TD M=104 ± 18.3		ABIDE	SVM	Atypical connectivity within DMN and salience network in both ASD and EOP. Distinct atypical connectivity for ASD was within-salience network.
90	213 (91/122)	14.87(1.61)	15(1.61)	0%/0%		ASD IQ=107.45 ± 12.11 TD IQ=109.30 ± 11.08		ABIDE	DFC/SBA	Underconnectivity within-DMN regions, between DMN and visual as well as ventral attention network in lower frequency bands (slow-4, slow-5).
91	54 (28/26)	13.79(1.79)	14.46(1.45)	17.9%/19.2%		ASD IQ=108.06 ± 13.86 TD IQ=110.11 ± 7.87		ABIDE	ALFF	Mixed results with lower ALFF values in rPCUN hub of DMN, and higher ALFF values in mPFC hub of DMN only for adolescents.
92	31 (15/16)	21.6(3.7)	21.9(3.5)	0%/0%		ASD IQ=111 ± 10 TD IQ=123 ± 9.2			SBA	Mixed results with underconnectivity between mPFC hub of DMN and bilateral AG region, and overconnectivity between DMN coupling with task positive fusiform face are (FFA) and supramarginal gyri (SMG).
93	92 (50/42)	13.34(2.41)	13.05(1.82)	10%/14.3%)		ASD IQ=99.73 ± 14.40 TD IQ=107.21 ± 10.94		ABIDE	ICA	Mixed results with underconnectivity within anterior hubs of the DMN of mPFC, inferior frontal gyrus-triangularis (IFGtriang) and overconnectivity with posterior hubs of the DMN (PreCG, SPG, PCUN).
94	102 (49/53)	17.39(3.1)	16.8(2.95)	12.2%/23.3%		ASD IQ=103.65 ± 14.46 TD IQ=108.81 ± 10.76			SBA	Overconnectivity between DMN and salience as well as frontoparietal network.
95	718 (369/349)	13.53	13.54	0%/0%		Not reported		ABIDE	SVM	Underconnectivity between DMN and salience network and lower coupling of DMN and right temporoparietal junction (rTPJ) node of dorsal attention network.
96	150 (62/10)	16.16(1.21)	16.16(1.21)	45.2% F		ASD IQ=100.5 ± 16.05			SBA	Underconnectivity between DMN (PCC, vmPFC) and salience network (ACC, rAI) hubs in adolescents.
97	51 (22/29)	17.45(3.29)	18.48(2.82)	18.2%/34.5%		ASD IQ=99.77 ± 9.5 TD IQ=105.83 ± 9.64			SBA	Mixed results with underconnectivity between PCC hub and executive control component of DMN (ACC, IFG, SFG, middle temporal regions), and overconnectivity between mPFC hub and sensorimotor component of DMN (amPFC, bilateral Pre-and Post-CG).
98	98 (49/49)	14.35(1.77)	14.35(1.77)	0%/0%		Not reported but groups matched for IQ ( ± 10 points)		ABIDE	SBA	Underconnectivity between rPCUN hub of DMN and right middle temporal gyrus (rMTG) as well as bilateral Post-CG.
99	507 (209/298)	16.5(6.2)	16.8(6.2)	0%/0%		ASD IQ=110.6 ± 13.4 TD IQ=110.2 ± 11.4		ABIDE	DFC/SBA	Underconnectivity within vmPFC and PCC hubs of the DMN with rAI in social cognition dynamic states (state 3, state 5).
100 (Time 1)	38 (16/22)	12.5(0.8)	12.9(0.9)	6.3%/0%		ASD IQ=101.3 ± 17.7 TD IQ=107.8 ± 13.5		ABIDE II	SBA	Atypical developmental trajectory with lower negative connectivity between DMN and central executive network longitudinally from early to late adolescence.
100 (Time 2)	38 (16/22)	15.5(0.8)	16.0(0.9)	6.3%/0%			
101	119 (62/57)	13.7(2.5)	13.1(2.9)	16.1%/19.3%		ASD IQ=103 ± 18 TD IQ=108 ± 12			DFC/ICA	Overconnectivity between DMN and visual, sensorimotor, frontoparietal, and executive network in static state; along with increased variability in DMN across dynamic states.
102	88 (44/44)	11.2(2.7)	10.9(2.8)	~22%/~22%		Low ASD IQ=77 ± 6 High ASD IQ=123 ± 8 Average TD IQ=99 ± 7 High TD IQ=124 ± 8		ABIDE II	SBA	Underconnectivity within-DMN in lower-functioning participants more prominent than higher-functioning participants.
103	260 (83/177)	11.2(5.3)	11(4)	16.9%/24.9%		No Full-Scale IQ ASD NVIQ=105.0 ± 15.7 TD IQ=106.1 ± 11.2		ABIDE II	ICA	Mixed results with underconnectivity within-DMN regions, and overconnectivity between DMN and somatomotor network.
104	102 (52/50)	13.7(2.6)	13.6(2.6)	15.38%/16%		ASD IQ=104 ± 16.4 TD IQ=107 ± 11			SBA	Overconnectivity between PCC hub of DMN and IFG and visual cortex bilaterally

**Table 2:** Summary of demographic details and results for studies on DMN functional connectivity in adolescents with EOP.

Study	Sample Size	Age		Sex (F%)	IQ	Patient Characteristics	Analysis	Results for EOP group
	N (EOP/TD)	EOP Mean (SD)	TD Mean (SD)	EOP/TD				
105	64 (32/32)	16.2(1.2)	16.4(0.9)	53.1%/50%	EOP=9.4 ± 1.5 TD=9.7 ± 0.7	Youth with first-episode schizophrenia < 2 years illness onset	ICA/ALFF	Overconnectivity between mPFC and other areas of the DMN.
106	67 (37/30)	15.5(1.8)	15.3(1.6)	54.1%/43.3%	EOP=8.5 ± 1.48 TD=8.7 ± 1.42 IQ > 70 both groups	Drug-naïve patients with first-episode schizophrenia < 2 years illness onset	SBA	Underconnectivity between rMTG seed region of DMN and lITG, lFFA, lPHG, as well as between DMN and visual network regions.
107	102 (31/37) UHR 34	20.61(4.42) UHR 21.50(3.53)	20.76(3.08)	38.7%/51.4% UHR 38.2%	EOP=6.26 ± 4.27 UHR =6.26 ± 4.13 TD=5.46 ± 1.87	Drug-naïve patients with first-episode schizophrenia < 2 years illness onset UHR included brief intermittent psychotic syndrome, attenuated positive symptom syndrome, and genetic risk and deterioration syndrome	SBA	Overconnectivity between DMN (PCUN/PCC, mPFC) and cerebellum in both EOP and UHR groups; with UHR group showing stronger patterns of cerebellar-DMN connectivity than EOP group.
108	65 (35/30)	15.5(1.8)	15.3(1.6)	42.9%/56.7%	EOP=8.5 ± 1.48 TD=8.7 ± 1.42	Drug-naïve patients with first-episode schizophrenia < 2 years illness onset	SBA/ALFF	Lower ALFF values in vPCUN, along with underconnectivity between vPCUN and dPCUN as well as midcingulate cortex (MCC).
89	66 (35/31)	15.6(1.8)	15.4(1.6)	42.86%/58.06%	Not Reported	Drug-naïve patients with first-episode schizophrenia < 2 years illness onset	SVM	Atypical connectivity within DMN and salience network in both EOP and ASD. Distinct atypical connectivity for EOP was between DMN-salience connectivity.
109	51 (32/19)	AVH 21.24(3.85) Non-AVH 22.53(4.07)	23.79(3.75)	AVH 41.18% Non-AVH 46.67% TD 47.37%	AVH=13.71 ± 1.93 Non-AVH= 13.40 ± 1.55 TD=14.74 ± 2.26	Patients with schizophrenia experiencing AVHs vs. Patients with schizophrenia not experiencing AVHs	ICA/ALFF	Higher signal amplitude within-DMN regions (mPFC, ACC, PCC, AG, rSPG) along with increased prefrontal cortex-DMN coactivation in patients with AVHs versus non-AVH patients. AVH patients also demonstrated more atypical ALFF values in PCUN than non-AVH patients.
110	65 (35/30)	15.5(1.8)	15.3(1.6)	42.9%/56.7%	EOP=8.5 ± 1.48 TD=8.7 ± 1.42	Drug-naïve patients with first-episode schizophrenia < 2 years illness onset	DFC	Underconnectivity in PCUN hub of DMN in slow-4 frequency band, but no significant group differences in slow-5 frequency band.
111	79 (48/31)	15.79(1.64)	15.42(1.52)	56.3%/54.8%	EOP=8.88 ± 1.95 TD=8.44 ± 1.56 IQ > 70 both groups	Drug-naïve patients with first-episode schizophrenia < 2 years illness onset	SVM/ReHo	Decreased ReHo values in rPre-CG, lPost-CG, rIPL, rMFG, bilateral PCUN, left superior temporal gyrus (lSTG), left paracentral lobule regions of the DMN. Reho values in bilateral PCUN and rIPL especially discriminated patients with 91.67% sensitivity, 87.1% specificity, and 89.87% accuracy.
112	79 (48/31)	15.79(1.64)	15.42(1.52)	56.3/54.8%	EOP=8.88 ± 1.95 TD=8.44 ± 1.56 IQ > 70 both groups	Drug-naïve patients with first-episode schizophrenia < 2 years illness onset	SVM	Mixed results with underconnectivity of both long- and short-range networks involving posterior DMN hubs, and overconnectivity of both long- and short -range networks involving anterior DMN hubs.
113	79 (48/31)	15.79(1.64)	15.42(1.52)	56.3/54.8%	EOP=8.88 ± 1.95 TD=8.44 ± 1.56	Drug-naïve patients with first-episode schizophrenia < 2 years illness onset	SVM/ReHo	Mixed results with increased ReHo values in mPFC hub of DMN, and decreased ReHo values in lSTG, rPre-CG, rIPL, and left paracentral lobule; this combination was able to discriminate patients from controls with the sensitivity of 88.24%, specificity of 91.89%, and accuracy of 90.14%.
114	86 (48/38)	AVH 24.32(8.46) Non-AVH 24.35(6.94)	25.44(7.52)	AVH 53.5% Non-AVH 50% TD 55.3%	AVH=11.29 ± 3.00 Non-AVH= 11.70 ± 2.60 TD=13.34 ± 3.58	Drug-naïve patients with first-episode schizophrenia experiencing AVHs vs. Drug-naïve patients with first-episode schizophrenia not experiencing AVHs < 2 years illness onset for both groups	GCA	Mixed results with underconnectivity between DMN hubs (mPFC, PCC) and left inferior temporal gyrus (lITG), lSTG, bilateral cingulate gyrus, bilateral thalamus, left insula, and left cerebellum, with overconnectivity between hub regions and left cingulate gyrus, right putamen, rMFG, right thalamus, and left cerebellum. AVH patients demonstrated underconnectivity between aMPFC and lITG, as well as PCC to left cerebellum, lITG, and rMFG compared to non-AVH patients.
115	65 (35/30)	15.5(1.8)	15.3(1.6)	42.9/56.7%	EOP=8.5 ± 1.48 TD=8.7 ± 1.42	Drug-naïve patients with first-episode schizophrenia < 2 years illness onset	DFC	Mixed results with underconnectivity between lPCUN hub of DMN and lMTG in state2, and overconnectivity in rPCUN, rSMG, and right putamen in state 4.
116	68 (27/41)	18.1(1.6)	17.8(1.6)	59.3%/56.1%	EOP=92.8 ± 15.7* TD=104.1 ± 9.8*	Youth with early onset psychosis including schizophrenia, schizoaffective disorder, major depressive disorder with psychotic features, bipolar spectrum disorders, and psychosis not otherwise specified < 2 years illness onset	ICA	Underconnectivity of mPFC hub of DMN in EOP group compared to TD controls, and connectivity additionally decreased with age in EOP where it increased with age in TD controls.
117	79 (48/31)	15.79(1.64)	15.42(1.52)	56.3%/54.8%	EOP=8.88 ± 1.95 TD=8.44 ± 1.56 IQ > 70 both groups	Drug-naïve patients with first-episode schizophrenia < 2 years illness onset	SVM/NH	Mixed results with higher NH values in left mPFC and lower NH values in bilateral PCC/PCUN in EOP group compared to TD controls.

*Study reported IQ scores instead of education in years for demographic characteristics.

## DMN Connectivity in Adolescents With ASD

The past few years have witnessed a proliferation of resting-state connectivity studies in adolescents with ASD, facilitated in part by the availability of open-access multinational datasets such as the Autism Brain Imaging Data Exchange (ABIDE; 118). Approximately 52% of the studies in adolescents with ASD presented in our review (**Table 1**) have utilized the ABIDE dataset to investigate DMN connectivity (81, 84, 86–91, 93, 95, 98–100, 102, 103), using a combination of traditional SBA, SVM, ICA, ALFF, and DFC analytic techniques. These studies appear to be using a largely overlapping, although not exactly the same, set of ABIDE participants. About half of the studies (15 out of 29) in adolescents with ASD have found a global pattern of underconnectivity both within the DMN hubs (77, 78, 80, 87, 88, 90, 96, 98, 102), as well as between the DMN and other brain regions such as insula, subcortical regions, fronto-parietal regions, and visual cortex (73, 81, 84, 90, 95, 99), regardless of analytic methods used. Relatively fewer studies (five out of 29) have observed over-connectivity between the DMN and task-positive regions within the fronto-parietal, visual, and sensorimotor regions, as well as the salience network (79, 86, 94, 101, 104). Some studies (nine out of 29) have additionally found mixed patterns involving under- and over-connectivity of ASD youth relative to typically developing (TD) controls, largely highlighting a pattern of within-DMN underconnectivity, with overconnectivity between DMN and other networks such as task-positive or sensorimotor networks (82, 83, 85, 89, 91–93, 97, 100, 103). Of the studies using the ABIDE dataset, one reported predominant DMN overconnectivity with task-positive regions (86), while nine collectively reported DMN underconnectivity both within its hubs (87, 88, 90, 98, 102) as well as with other brain regions (81, 84, 90, 95, 99). Another five studies using the ABIDE dataset reported mixed DMN connectivity findings (89, 91, 93, 100, 103) using a range of analytic techniques (see **Table 1** for main results from each study).

Additional perspectives on DMN connectivity in ASD have been offered by new and emerging studies investigating whole brain DFC. Functional connectivity of neural networks are not static (temporally nor spatially); hence, functional connectivity of a network can vary in terms of connectivity strength and directionality during different temporal “windows” or timepoints of a scan (67, 119), Additionally, DFC clustering analysis allows for the identification of recurring patterns of connectivity among networks that is consistent with those observed during tasks. This coupling of specific functional networks during various timepoints of a resting-state scan are often referred to as “states” or state-dependency” of neural activity (67, 120). While some of these studies have found broader temporal variability of DMN connectivity across states in adolescents with ASD (87, 101), others show predominant patterns of underconnectivity between the DMN and salience, attentional, and visual networks, which is state-dependent and may be related to social cognition states (90, 99). Since DFC is a relatively new realm of functional connectivity research, additional investigations of dynamic DMN connectivity as it relates to adolescents with ASD is warranted to further delineate such state-dependent patterns. In addition to static versus dynamic models, one study also examined lateralization of the DMN and its relationship to language networks in adolescents with ASD (81), and found that the ASD group had significantly less left lateralization of these networks compared to TD controls, suggesting that these language and social cognition networks may not be as functionally specialized in ASD. They additionally found that this reduced left-lateralization was associated with higher ASD symptom severity.

A few studies have also explored the maturational trajectory of DMN connectivity in individuals with ASD (73, 84, 100). Wiggins et al. (73) looked at age-related patterns of DMN connectivity cross-sectionally, and found that the ASD group did not demonstrate typical age-related increases in connectivity between the precuneus/PCC hub of the DMN and frontal regions. Nomi and Uddin (84) further looked at differences in DMN connectivity between children and adolescents with ASD cross-sectionally, and found that children with ASD showed a pattern of within-DMN overconnectivity and between-network DMN underconnectivity relative to controls. Comparatively, adolescents with ASD did not differ from age-matched controls in within-DMN connectivity but demonstrated underconnectivity between DMN and the salience network and subcortical regions. In a longitudinal study, Lawrence et al. (100) looked at changes in DMN connectivity between ASD and TD controls from early to late adolescence, and found that TD controls had an age-associated increase in *negative* functional connectivity between the DMN and the task-positive central executive network, not observed in adolescents with ASD. These findings support the theory of a crucial maturational shift in DMN connectivity patterns during adolescence which is likely significantly impacted in individuals with ASD such that the typically expected strengthening and honing of DMN connectivity is disrupted in this population during this age period. However, the mechanism underlying the shift in DMN connectivity patterns after the onset of puberty is not fully understood in ASD yet, and requires further exploration to elucidate differential trajectories and their impact on symptomatology.

So far, only one study has systematically examined sex differences in DMN connectivity in ASD (88). This study spanned a wide age range from childhood to adulthood, but in the adolescent subset female TD controls demonstrated stronger within-DMN connectivity relative to male TD controls; comparatively, ASD females and males showed similar within-DMN connectivity strength, that in turn was significantly lower than their TD counterparts. Notably, this DMN hypoconnectivity appeared to be an endophenotype, as it was also observed in the unaffected siblings of ASD cases, relative to TD controls. These findings suggest aberrant DMN connectivity may underlie a broader continuum of autism-relevant traits in the general population.

Intellectual functioning is another variable of interest relevant to DMN connectivity in adolescents with ASD, given the wide range of cognitive abilities in this population (121). Most of the studies included in this review focused on adolescents within the normative intellectual functioning range; however, one recent study (101) examined the differences in within-DMN connectivity between low (Mean IQ=77 ± 6) and high-IQ ASD participants (Mean IQ=123 ± 8) and found that the low cognitive functioning group demonstrated significant within-DMN underconnectivity compared to the high-functioning group, even after controlling for symptom severity.

Lastly, several of the studies (12 out of 29) included in our review have examined the relationship between aberrant DMN connectivity in adolescents with ASD and behavioral measures of symptom severity such as the Autism Diagnostic Observation Schedule (ADOS; 122, 123), the Autism Diagnostic Interview-Revised (ADI-R; 124), and the Social Responsiveness Scale (SRS; 125, 126). Studies looking at the association of within-DMN network connectivity with behavioral measures of symptom severity (N=6 studies) found mixed effects, with most (five out of six studies) reporting greater within-DMN network underconnectivity associated with higher social impairment scores on the ADOS (77, 90, 91), ADI-R (78, 103), and SRS (77); while one of the six found greater within-DMN overconnectivity to be associated with higher social impairment scores on the SRS (83), and one (using the ADOS) reporting a mixed pattern (91). Studies looking at the association of DMN between-network connectivity with behavioral measures of symptom severity (N=7) mostly found that greater overconnectivity between DMN and other brain regions (mostly in the frontal and temporal lobes; four out of seven studies) was associated with higher social impairments on the ADOS (89), ADI-R (trend level; 97), and SRS (85, 86). Interestingly, in one of the first studies to report DMN overconnectivity in adolescents with ASD, Redcay et al. (79) found that greater DMN overconnectivity with the right lateral parietal region was associated with less impairment on the social-communication domain of the ADOS, suggesting the possibility of an underlying compensatory mechanism in this particular brain network. Only two out of seven studies looking at the association of DMN between-network connectivity with behavioral measures of symptom severity found that greater underconnectivity between DMN hubs and other brain regions (salience, attention networks) in ASD was associated with higher symptom severity on the ADOS (90, 99). Additionally, Doyle-Thomas et al. (82) and Ypma et al. (88) found that anomalous DMN connectivity patterns in adolescents with ASD (mixed within-DMN connectivity results in the former study, and within-DMN underconnectivity in the latter study) were associated with poorer performance on the “Reading the Mind in the Eyes” Test (RMET; 127), a measure of affective theory of mind (ToM). Of the studies (all 12 reporting associations between DMN connectivity and behavioral measures of symptom severity) that looked at both the social interaction domain and the repetitive behaviors/restricted interests domain of the ADOS-2 and ADI-R (77–79, 83, 85, 86, 89–91, 97, 99, 103), only one study (78) reported a significant relationship for DMN within-network underconnectivity patterns and measures of repetitive behaviors/restricted interests (ADI-R) in ASD adolescents. Hence, it appears that aberrant DMN connectivity may play a larger role in the social functioning deficits experienced by this population rather than other features of ASD.

## DMN Connectivity in Adolescents With EOP

Prior research on adults with schizophrenia spectrum disorders suggests that disrupted DMN connectivity may play an important role in the pathophysiology of schizophrenia (63). Specifically, findings in adults with schizophrenia frequently include within-DMN overconnectivity, as well as mixed findings of under- and over-connectivity between DMN and task-positive networks; in turn, these disruptions have been associated with positive symptoms, poor social functioning, as well as poor cognitive functioning in schizophrenia (63). Additionally, DMN connectivity has been found to become more “normative” in response to anti-psychotic treatment in adults with schizophrenia (128, 129). Some of the inconsistencies found in the literature on DMN connectivity patterns in schizophrenia, with both under- and over-connectivity involving this network being associated with symptom severity, as well as social and cognitive functioning, could be attributed to the heterogeneity of patient characteristics within schizophrenia spectrum disorders. For instance, studies thus far have included individuals with first-episode schizophrenia, chronic patients, drug-naïve patients, as well as patients treated with antipsychotic medications which may have impacted the results across these studies. Hence, how disease progression as well as treatment status impacts DMN connectivity and its relationship with behavioral outcomes in schizophrenia is not yet clear.

More recent studies of DMN connectivity in adolescents with EOP offer some insight into neural anomalies in the earlier stages of this disorder (**Table 2**). Several EOP studies (11 out of 14) have focused on drug-naïve adolescent patients with psychotic disorder with illness onset within two years (89, 106–108, 110–115, 117). Of these, the majority (eight out of 11) appear to report on the same (or at least, largely overlapping) cohort (106, 108, 110–113, 115, 117). Other EOP studies (three out of 14) have involved independent cohorts of adolescents with recent-onset psychotic disorder receiving anti-psychotic treatment (105, 109, 116). Collectively, results suggest a mixed pattern of under- and over-connectivity involving the DMN, similar to that observed in adults with schizophrenia (63) and regardless of analytic method or cohort used (see **Table 2** for main results from each study).

One study comparing adolescents at clinical high risk for psychosis (CHR) to drug-naïve adolescents with a diagnosed psychotic disorder suggested that while both groups showed increased connectivity between DMN and cerebellum compared to TD control groups, the connectivity strength was attenuated in those with overt illness (107). On the other hand, some studies of drug-naïve adolescents with psychotic disorder have reported underconnectivity within the DMN (108, 110, 111) relative to healthy controls, and between DMN and other brain areas such as prefrontal cortex, temporal gyrus, parietal cortex, and limbic regions (106). However, six out of 14 studies investigating DMN connectivity in youth with EOP indicate a mixed pattern of connectivity, both within the DMN as well as between the DMN and other brain regions such as temporal lobe, subcortical regions, and cerebellum (89, 112–115, 117). One interesting perspective offered by Wang et al. (112) from their examination of short versus long-range DMN connectivity is that there is potentially a pattern of overconnectivity involving the anterior hubs of the DMN, compared to underconnectivity involving the posterior hubs of the DMN in drug-naïve adolescents with psychotic disorder. This perspective is further supported by recent findings of higher network homogeneity in anterior hubs of the DMN but lower in posterior hubs of the DMN in drug-naïve adolescents with psychotic disorder compared to controls (117). In the past few years, studies investigating whole-brain DFC in adolescents with EOP have also emerged (110, 115). These studies have been largely consistent with the mixed connectivity findings of the DMN for drug-naïve adolescents with a diagnosed psychotic disorder (110, 115) and suggested that over- or under-connectivity of the DMN could be state-dependent, with the precuneus hub of the DMN especially demonstrating differential state-dependent connectivity patterns with other brain regions.

Studies of youth with EOP receiving anti-psychotic medication mostly showed overconnectivity relative to healthy controls within the DMN (105, 109), as well as increased co-activation between DMN and prefrontal cognitive control regions (109) with only one study reporting underconnectivity within the DMN (116). It is therefore tempting to speculate that prior to the introduction of anti-psychotic medication the DMN tends to be underconnected or mixed in its connectivity patterns, with changes occurring in the pattern of connectivity after implementation of a medication regimen or as the course of the disease progresses.

Current symptom severity may also impact DMN connectivity patterns in adolescents with EOP. Ten out of 14 studies reviewed here examined the relationship between DMN connectivity and symptom severity on the Positive and Negative Syndrome Scale (PANSS), a widely used measure in schizophrenia (130). Out of these, four studies did not find any significant associations between DMN connectivity patterns and PANSS scores (89, 112, 115, 116). However, results from six out of 10 studies that found significant relationships between DMN connectivity and the PANSS revealed that aberrant within-DMN connectivity (105, 108, 110) as well as disrupted connectivity between DMN and other brain regions (106, 107, 114) in EOP tended to be more strongly associated with PANSS negative symptoms scores rather than positive symptom scores. Lastly, one recent study found that within-DMN underconnectivity accounted for ~16% of the variance in ToM performance measured by the RMET in adolescents with EOP treated with anti-psychotics (116). This suggests that the DMN may have a more crucial role in the social impairments observed in adolescents with EOP, rather than positive symptoms such as unusual thought content or perceptual disturbances.

## Shared and Distinct DMN Connectivity Patterns in Adolescents With ASD and EOP

**Figure 1:**
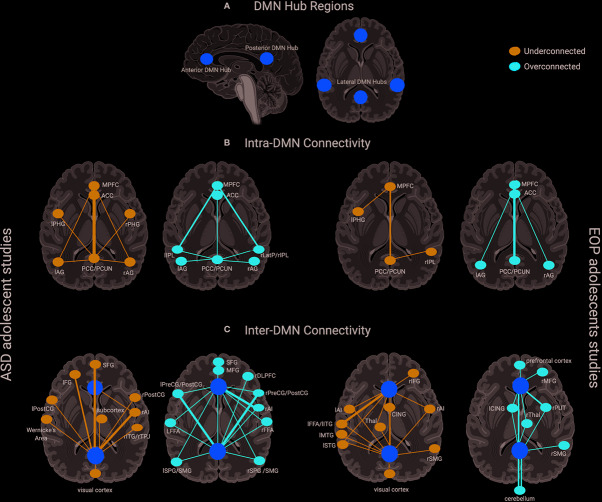
**(A)** DMN hub regions included in analyses across most studies in the present review denoted in yellow circles—anterior hubs include medial prefrontal regions (mPFC) and anterior cingulate cortex (ACC), posterior hubs include the posterior cingulate cortex (PCC) and precuneus (PCUN), lateral hubs include inferior parietal lobule (IPL), angular gyrus (AG), and medial temporal regions such as parahippocampal gyrus (PHG); **(B)** Intra-DMN connectivity findings across ASD adolescent studies (left panel)—Underconnectivity findings (denoted by blue dots) mostly involve the posterior hubs of the DMN. Thicker lines such as between the PCC/PCUN and the anterior hubs of the DMN (mPFC, ACC) denote overlapping findings across multiple studies, with thinner lines indicating underconnectivity within DMN regions found in fewer studies. Overconnectivity findings (denoted by red dots) for the ASD group involve the anterior hubs of the DMN slightly more prominently than the posterior hubs on the DMN. Thicker lines such as between the mPFC and left and right IPL denote overlapping findings across multiple studies, with thinner lines indicating overconnectivity within DMN regions found in fewer studies. Intra-DMN connectivity findings for EOP adolescent group are depicted in the right panel—Underconnectivity within the DMN regions (blue dots) was found by fewer studies for this group indicated by thinner lines, while overconnectivity (red dots) was mostly found within the anterior and posterior hubs of the DMN; **(C)** Inter-DMN connectivity findings across ASD adolescent studies (left panel)—Underconnectivity findings (denoted by blue dots) mostly involve the posterior hub (PCC/PCUN) of the DMN especially in its connectivity with prefrontal regions and the right anterior insula (rAI) hub of the salience network denoted by thicker lines with additional findings of underconnectivity between DMN and other brain regions denoted by thinner lines. Overconnectivity findings (denoted by red dots) for the ASD group also involve the posterior hubs of the DMN mostly with somatomotor regions as well as anterior hubs of the DMN with the salience network hub (rAI) denoted by thicker lines. Other regions demonstrating overconnectivity with the DMN for the ASD group are denoted by thinner lines. Inter-DMN connectivity findings for EOP adolescent group are depicted in the left panel—Underconnectivity between anterior DMN hubs and left temporal lobe was most prominently found across studies depicted by thicker lines, with additional findings of underconnectivity between both the anterior and posterior hubs of the DMN and other brain regions denoted by thinner lines. On the other hand, overconnectivity in the EOP group was predominant between DMN hubs (both anterior and posterior) and the subcortex and cerebellum highlighted by thicker lines, with additional overconnectivity between DMN hubs and other brain regions denoted by thinner lines. Additional abbreviations used in figure: PHG, parahippocampal gyrus; AG, angular gyrus; LatP, lateral parietal lobule; SFG, superior frontal gyrus; IFG, inferior frontal gyrus; PreCG, precentral gyrus; POSTCG, postcentral gyrus; ITG, inferior temporal gyrus; TPJ, temporoparietal junction; MFG, middle frontal gyrus; DLPFC, dorsolateral prefrontal gyrus; FFA, fusiform face area; SPG, superior parietal gyrus; SMG, supramarginal gyrus; CING, cingulate gyrus; Thal, thalamus; MTG, middle temporal gyrus; STG, superior temporal gyrus; PUT, putamen.

**Figure 1** provides a schematic representation of findings from all the studies for each group, with yellow dots representing the DMN hub regions, blue (underconnected) or red (overconnected) dots representing connectivity with other brain regions, and thickness of lines connecting the dots representing frequency of findings across studies in each group. Here, we see that studies examining within-DMN connectivity (intra-DMN) found underconnectivity involving the posterior hub of the DMN or between the anterior and posterior hubs of the DMN more frequently in ASD (77, 78, 80, 82, 83, 85, 87, 88, 90, 91, 97, 102, 103), while overconnectivity involving the anterior hub or between the anterior and posterior hubs of the DMN was often found in EOP studies (105, 109, 112, 113, 117). Some studies reported intra-DMN underconnectivity in EOP involving the posterior hub of the DMN or between the medial and lateral hubs of the DMN (106, 108, 110, 117). However, it should be noted that all these studies reporting intra-DMN underconnectivity in EOP are based on the same or largely overlapping subjects. On the other hand, overconnectivity within the ASD group was most frequently seen between the anterior and lateral hubs of the DMN (79, 85, 86, 91, 97). For studies examining connectivity between DMN and other brain regions (inter-DMN), underconnectivity in ASD relative to TD controls mostly involved the posterior hub of the DMN and frontal regions as well as right anterior insula, a hub region of the salience network (73, 82, 84, 89, 93, 95–99). It should be noted that six out of 10 of these studies reporting inter-DMN underconnectivity involving the posterior hub in ASD are based on the same or largely overlapping ABIDE subjects (84, 89, 93, 95, 98, 99); as such, these cannot be considered independent findings. In contrast, inter-DMN underconnectivity for EOP relative to controls was seen most frequently between the anterior hub of the DMN and left temporal lobe (106, 111, 113–115). Overconnectivity for inter-DMN networks in the ASD group also involved the posterior hubs of the DMN, mostly with somatomotor and visual regions as well as anterior hubs of the DMN with the right anterior insula (82, 85, 86, 92–94, 97, 100, 101, 103, 104). In contrast, inter-DMN overconnectivity in EOP relative to controls was predominantly observed between DMN hubs (both anterior and posterior) and the subcortex and cerebellum (107, 114, 115). Hence, it appears that intra-DMN networks seem to be more frequently underconnected (between anterior and posterior hubs) in ASD adolescents, but mixed (i.e., underconnected for anterior hub, or between medial and lateral hubs, and overconnected for posterior hub or between anterior and posterior hubs) in EOP adolescents. On the other hand, inter-DMN connectivity patterns appear to be mixed for both groups, especially in its connectivity with frontal, sensorimotor, and temporoparietal regions in ASD, and with frontal, temporal, subcortical, and cerebellar regions in EOP.

Taken together, the findings reviewed thus far highlight that ASD and EOP have both convergent, as well as divergent, patterns of dysregulation of DMN networks. Convergently, the mixed findings reported to date suggest poor functional segregation and integration of the DMN in both ASD and EOP during adolescence. The only currently published study that has directly compared whole-brain connectivity patterns in adolescents with ASD and EOP (89) found that ASD and EOP youth shared a common pattern of disrupted connectivity compared to TD controls, mainly involving the prefrontal nodes of the DMN and salience networks, which is also implicated in social functioning (131, 132). In contrast, they found that disrupted connectivity *between* DMN and salience network was more characteristic of EOP, whereas in ASD the atypical connections were primarily found *within* the salience network. Studies examining the maturational trajectory of resting state networks in ASD suggest that DMN connectivity appears to decrease from childhood to adolescence (73, 84, 100), similar to the DMN “network pruning” found in studies of typically developing adolescents (36, 37, 41, 45). However, unlike typically developing adolescents, there is a lack of both strengthening between anterior and posterior hubs of the DMN and segregation from other brain regions reported during this developmental period in the reviewed ASD studies. There were no available studies examining longitudinal trajectories of DMN connectivity in EOP adolescents to address age-associated changes or medication effects in this group. Thus, more longitudinal studies are warranted to understand DMN connectivity changes as a function of development and disease progression in both ASD and EOP groups.

Additionally, the studies reviewed here that reported brain-behavior associations have mostly used symptom severity measures (such as the ADOS, ADI-R, SRS, PANSS) rather than measures of social functioning or social cognition per se. One reason for the absence of specific social cognition measures across studies might be the use of shared datasets which provides researchers with large sample sizes, but a limited amount of common behavioral data collected across all contributing sites. Another reason could be the absence of *a priori* hypotheses about the association between DMN connectivity and social functioning. Only two ASD studies (82, 88) and one EOP study (116) used the RMET to measure social cognition in these clinical populations. Notably, all three studies found a significant inverse association between intra-DMN connectivity and RMET performance in both patient groups (i.e., less intra-DMN connectivity was associated with poorer task performance). This might suggest that the strengthening of connectivity between anterior and posterior DMN hubs during adolescence plays a crucial role in social functioning, and disruptions to this process in these neuropsychiatric conditions may be pertinent to social impairments. Other studies reporting brain-behavior findings additionally suggest that disrupted DMN connectivity appears to be associated with social impairments in both ASD (using the ADOS, ADI-R, or SRS) and EOP (using the PANSS), rather than other features distinct to each condition (e.g., repetitive behaviors and restricted interests in ASD vs. presence of hallucinations or delusions in EOP). Collectively, these may be considered preliminary findings for the shared role of DMN connectivity specifically underlying social functioning deficits characteristic of both ASD and EOP. However, more comprehensive assessments of social cognition abilities are needed in future studies to better elucidate the shared and distinct role of DMN connectivity disruptions in the developing brain and its relevance to social functioning.

## Future Directions

In this review, we have summarized resting state functional MRI studies of DMN connectivity from empirical studies in two different clinical populations involving marked social impairment, autism spectrum disorder and early onset psychosis, during the crucial developmental window of adolescence. While the literature thus far has helped shed some light on both the common and unique patterns of DMN connectivity across these two groups, several gaps remain in our understanding of how DMN connectivity might contribute to the unique pathophysiology of both neuropsychiatric conditions. First, there have been far fewer studies on DMN connectivity in EOP than ASD adolescents. This may be due in part to difficulties in ascertaining adolescents with EOP compared to adults with psychotic disorder, given its relatively lower prevalence (133, 134). Another reason is the wider availability of large, open-access imaging datasets of adolescents with ASD such as ABIDE. Given the difficulties of collecting neuroimaging data in unique clinical populations at single sites, it is advantageous for more researchers to combine their imaging datasets using a systematic and open-source forum to allow large-scale statistical analyses cross-diagnostically. However, at present, the extent of overlap in subjects is unclear in ASD studies using ABIDE data, as well as the EOP studies that used a similar cohort of drug-naïve adolescent patients. Additionally, differences in methodologies implemented across ASD and EOP studies preclude direct comparisons of effect size for the reviewed findings. Hence, we encourage future studies on DMN connectivity in these patient populations to provide greater transparency and consistency in reporting of methods and results. Furthermore, both these conditions are characterized as spectrum disorders of varying severity, heterogeneous etiologies, and comorbidities. Since the DMN connectivity patterns observed for each of these neuropsychiatric disorders did not appear to map on well to the symptom severity measures used to assess social functioning (ADOS, ADI-R, SRS, PANSS), future studies might want to examine dimensional relationships between symptoms, social cognition performance, and their neural correlates, rather than the traditional case-control designs implemented in the majority of studies to date. Such a dimensional approach can include multiple neural features of DMN connectivity and more detailed clinical assessments of social cognition, but will require larger samples to provide meaningful findings. Finally, the impact of factors such as genetic risk, symptoms endorsed, pubertal development, and treatment history on DMN connectivity have yet to be explored within both groups. For instance, few studies have examined the contribution of medication on DMN connectivity, despite evidence that antipsychotic medication can impact brain connectivity patterns (63, 128, 135). Similarly, more work examining the impact of disease progression in EOP on DMN connectivity is needed to understand if abnormal DMN connectivity within this population remains relatively stable across the duration of illness or if further declines are associated with longer-term illness. In the ASD population, imaging studies have generally focused on high-functioning individuals, with only one study so far exploring the differences in DMN connectivity between high-and low-functioning ASD adolescents (101). It would be important to explore the influence of such contributing factors to DMN connectivity anomalies to interpret the divergent findings across studies and develop a potential mechanistic model of how genetics, neural wiring, and environmental factors may cascade into the phenotypic features we observe in these neuropsychiatric conditions.

## Author Contributions

AN and CB took the lead role in reviewing papers and drafting the manuscript. MJ and SL assisted in reviewing papers and preparing tables. All authors read and approved the final manuscript. All authors contributed to the article and approved the submitted version.

## Funding

This work was supported by the National Institute of Mental Health K99MH113820 (author AN).

## Conflict of Interest

The authors declare that the research was conducted in the absence of any commercial or financial relationships that could be construed as a potential conflict of interest.
